# Oral health-related quality of life after dental general anaesthesia treatment among children: a follow-up study

**DOI:** 10.1186/1472-6831-14-81

**Published:** 2014-07-01

**Authors:** Birute Jankauskiene, Jorma I Virtanen, Ricardas Kubilius, Julija Narbutaite

**Affiliations:** 1 Clinic for Preventive and Paediatric Dentistry, Lithuanian University of Health Sciences, Lukšos - Daumanto 6, LT - 50106 Kaunas, Lithuania; 2 Institute of Dentistry, University of Oulu, Oulu, Finland; 3 Oral and Maxillofacial Department, Oulu University Hospital, Oulu, Finland; 4 Clinic for Maxillofacial Surgery, Lithuanian University of Health Sciences, Kaunas, Lithuania; 5 Lithuanian University of Health Sciences Hospital, Kaunas, Lithuania

**Keywords:** Dental general anaesthesia, Oral health-related quality of life, Children, Follow-up

## Abstract

**Background:**

Many young paediatric patients with severe dental caries receive dental treatment under general anaesthesia. Oral health-related quality of life (OHRQoL) can be evaluated to assess the outcome of dental general anaesthesia (DGA) treatment. The aim of our study was to examine the OHRQoL of young Lithuanian children in need of DGA treatment and analyse the impact of DGA treatment on children’s OHRQoL.

**Methods:**

We carried out a prospective clinical follow-up study on OHRQoL among all young Lithuanian child patients who received DGA treatment at the Lithuanian University of Health Sciences Hospital during 2010–2012. The study consisted of clinical dental examinations of patients younger than six years, data collected from their patient files, and an OHRQoL survey completed by their parents prior to the child’s dental treatment. We conducted a follow-up OHRQoL survey one month after the DGA treatment. The Early Childhood Oral Health Impact Scale (ECOHIS) and its effect size (ES) served to evaluate children’s OHRQoL, and the Wilcoxon signed-rank test served for statistical analyses.

**Results:**

We obtained complete baseline and follow-up data for 140 and 122 participants, respectively (84.7% follow-up rate). Pain and eating problems among children and parents feeling upset and guilty were the most frequently reported impacts at baseline. The parents reported greater impacts on boys than on girls. The ECOHIS score decreased significantly (69.5%, p < 0.001) after DGA treatment, revealing a large ES for the child (1.6) and family (2.4) sections of the ECOHIS.

**Conclusions:**

The OHRQoL of young Lithuanian children requiring DGA treatment is seriously impaired. Dental general anaesthesia treatment results in significant improvement of the children’s OHRQoL. The children’s parents also greatly appreciate this treatment modality for its positive impact on the family’s quality of life.

## Background

Early childhood caries (ECC) is one of the most common health problems among toddlers and preschool-age children [1]. Although the majority of children are able to receive dental treatment in a conventional setting, some patients fail to respond to the usual behaviour management techniques and must therefore be treated under dental general anaesthesia (DGA). DGA treatment, though effective, poses risks to patients’ overall health [2]; it is also a costly and resource-intensive method and therefore requires clear evidence of its benefits for children and their families.

Assessing the outcome of full mouth rehabilitation under general anaesthesia (GA) requires an evaluation of children’s oral health-related quality of life (OHRQoL). Because DGA treatment is commonly performed in one session, measuring the effect of the treatment on a patient’s OHRQoL is possible. Several OHRQoL measures have been developed for use among children [3-7]. The Early Childhood Oral Health Impact Scale (ECOHIS) is the one designed for children of preschool age and younger [6] and recently short-form versions of the Parental-Caregiver Perceptions Questionnaire (P-CPQ) and the Family Impact Scale (FIS) have been introduced [7]. The original English version of the ECOHIS has been translated into other languages and has been used successfully in different countries [8-13]. Furthermore, it has also been found to be sensitive and responsive to DGA treatment effects [14].

Recent studies of the impact of DGA treatment on children’s OHRQoL have shown significant improvement in oral health and psychological, social and overall wellbeing as well as a positive impact on the family [14-19]. The anticipated impact on OHRQoL may, however, depend on the state of oral health of people and the oral health services available to them. Among high-ECC subjects, children’s OHRQoL may dramatically improve after the treatment [14], while changes among children in a highly developed oral health care situation may be more subtle and distinctive [17]. The prevalence of ECC in Lithuania is very high in international terms [20-22], and a recent study [23] showed that oral health among young children who received DGA treatment was among the poorest worldwide [14,24-26]. The impact of dental caries and DGA treatment on the quality of life of Lithuanian children and their families has not yet been studied. The aim of our study was to examine the OHRQoL of young Lithuanian children in need of DGA treatment and analyse the impact of DGA treatment on children’s OHRQoL.

## Methods

We conducted a prospective clinical follow-up study of OHRQoL among Lithuanian child patients who received DGA treatment. The Kaunas Regional Research Ethics Committee approved the study (No. BE −2-19, Date: 04/11/2009).

The study included all patients younger than six years who received comprehensive dental treatment under general anaesthesia (GA) during a three year period in 2010–2012 at the Lithuanian University of Health Sciences (LUHS) Hospital in Kaunas, Lithuania. All the patients were referred to DGA treatment from the Clinic for Preventive and Paediatric Dentistry (LUHS) after a consultation appointment with a specialist in paediatric dentistry [23]. A total of 144 patients participated in the study (those with developmental disorders and general diseases were excluded). The study was voluntary, and the children’s parents provided their written informed consent. A detailed description of the participants appears elsewhere [23].

The study consisted of clinical dental examinations performed during DGA, an OHRQoL survey completed by the parents of the children at the time of treatment, and data extracted from the patients’ files. We conducted the follow-up OHRQoL survey of the parents one month after the children’s DGA treatment.

The personal background data included the children’s gender, age in months, parental education (university, college, secondary, primary), and area of residence (city, town, small town, village).

Each GA session included a clinical dental examination and full dental rehabilitation. We recorded the findings of the examination, diagnoses, data on dental treatment, and duration of GA. The dental index of decayed, missing and filled teeth (dmft) served as a measure of dental caries experience [27]. Protocols for clinical dental examination and DGA treatment are described in full and appear elsewhere [23].

### Quality of life survey

On the day of DGA at the hospital, each patient’s parent/caregiver received a self-administered questionnaire measuring OHRQoL. The questionnaire enquired about the child’s oral state and wellbeing over the past three months. The follow-up survey used the same questionnaire one month after DGA treatment: the patients’ parents were invited to the clinic for the child’s dental check-up and to participate in the follow-up survey related to the child’s oral condition after DGA treatment. If the parent/caregiver failed to come to the appointment, the questionnaire was mailed to them.

The survey tool for assessing children’s OHRQoL was the previously developed and pretested Lithuanian version of the ECOHIS [13]. The ECOHIS consists of 13 questions relevant to preschool-age children [6]. The survey questionnaire relies on parental ratings of the 13 items grouped in two main parts: the child impact section and the family impact section. The child impact section covers four domains: child symptoms (1 item), child functions (4 items), child psychology (2 items), and child self-image and social interaction (2 items). The family impact section covers two domains: parental distress (2 items) and family function (2 items). Each question asks about the frequency of an oral health-related problem and is scored on a scale from 0–5, as follows: never (score 0), hardly ever (score 1), occasionally (score 2), often (score 3), very often (score 4), don’t know (score 5).

Our questionnaire included two additional general questions about the oral health and general wellbeing of the child, as in the original ECOHIS [6], utilizing a Likert scale. The first general question included in both the baseline and follow-up surveys, “How would you rate the health of your child’s teeth, lips, jaws and mouth?”, had five answer options: ‘excellent’, ‘very good’, ‘good’, ‘fair’ or ‘poor’ (score 1–5). The second general question at baseline was a modification of the original ECOHIS: “How much does the condition of your child’s teeth, lips, jaws or mouth affect his/her overall wellbeing?”; its four response options were: ‘not at all’, ‘some’, ‘a lot’ or ‘very much’ (score 0–3). At the follow-up, parents were asked about any change in child’s overall wellbeing since the treatment; the three answer options were: ‘stayed the same’, ‘changed a little’ or ‘changed a lot’ (score 1–3). In addition, at the follow-up, three supplementary questions enquired about the parents’ satisfaction with the treatment itself, information provided prior to the treatment, and whether the parents would consider another DGA treatment if needed.

### Data analysis

We used the Statistical Package for Social Sciences program for Windows (SPSS, version 17) to analyse the data and used the ECOHIS scores at baseline to assess the internal consistency reliability and cross-sectional construct validity of the questionnaire. We calculated Cronbach’s alpha for the total scale as well as for the child and family sections of the ECOHIS. We then examined the associations between means of baseline scores and the response categories of the global ratings in order to assess the cross-sectional construct validity. The associations were examined by parametric and nonparametric ANOVA.

We categorised the age of the patients into two categories based on the maturity and distribution of the children: < 4 years and 4–6 years. Parental education was also dichotomised based on the distribution: high education (university) and others (college, secondary) (there were no parents with primary education).

To measure OHRQoL, we added up the item scores to create a total ECOHIS score; the higher the score, the greater the impact on quality of life. ‘Don’t know’ responses were recoded as missing. For those with up to 30% missing responses, we imputed a score for the missing items as the average of the remaining items of the questionnaire. Questionnaires with more than 30% missing responses were excluded from the analysis. We calculated the total scores for the whole ECOHIS, the child and family sections and the following domains: child symptoms, child function, child psychology, child social wellbeing, parent distress, and family function [6]. Because each domain and section contained different numbers of items, we also calculated standardized scores (scores/item) (total score divided by the number of questions in the domain/section).

We then determined the magnitude of change in OHRQoL after DGA treatment by subtracting the ECOHIS scores at follow-up from those at baseline. The same calculations were made for the child and family sections as well as all the domains of ECOHIS. The Wilcoxon signed-rank test served to compare baseline and follow-up scores and test the statistical significance of the changes. The effect size was calculated by dividing the mean of change score by the standard deviation of the baseline score [28]. An effect of < 0.2 indicated a small, but clinically meaningful magnitude of change, 0.2-0.7 a moderate change and > 0.7 a large change.

## Results

A total of 144 children (79 boys and 65 girls) younger than six years (mean age 3.9 (SD 0.8) years, range: 25–71 months) received DGA treatment in 2010–2012 at the LUHS Hospital. The children’s mean dmft prior to the treatment was 12.9 (SD 3.5). All participated in the study, but four patients were excluded from the OHRQoL analysis due to having more than 30% missing answers in the baseline survey. The questionnaires were completed by mothers (93 %), fathers (5.6%) and other family members (grandmothers) (1.4%). In most cases (98.4%), the same respondent completed both the baseline and follow-up questionnaires. Of the 140 participants with complete baseline data, 23 failed to come to the follow-up appointment. The questionnaires were mailed to them and five were returned. Overall, we obtained a complete data set for 122 (84.7%) patients in the follow-up. There were no statistically significant differences in baseline characteristics between those retained and those lost.

**Figure 1:**
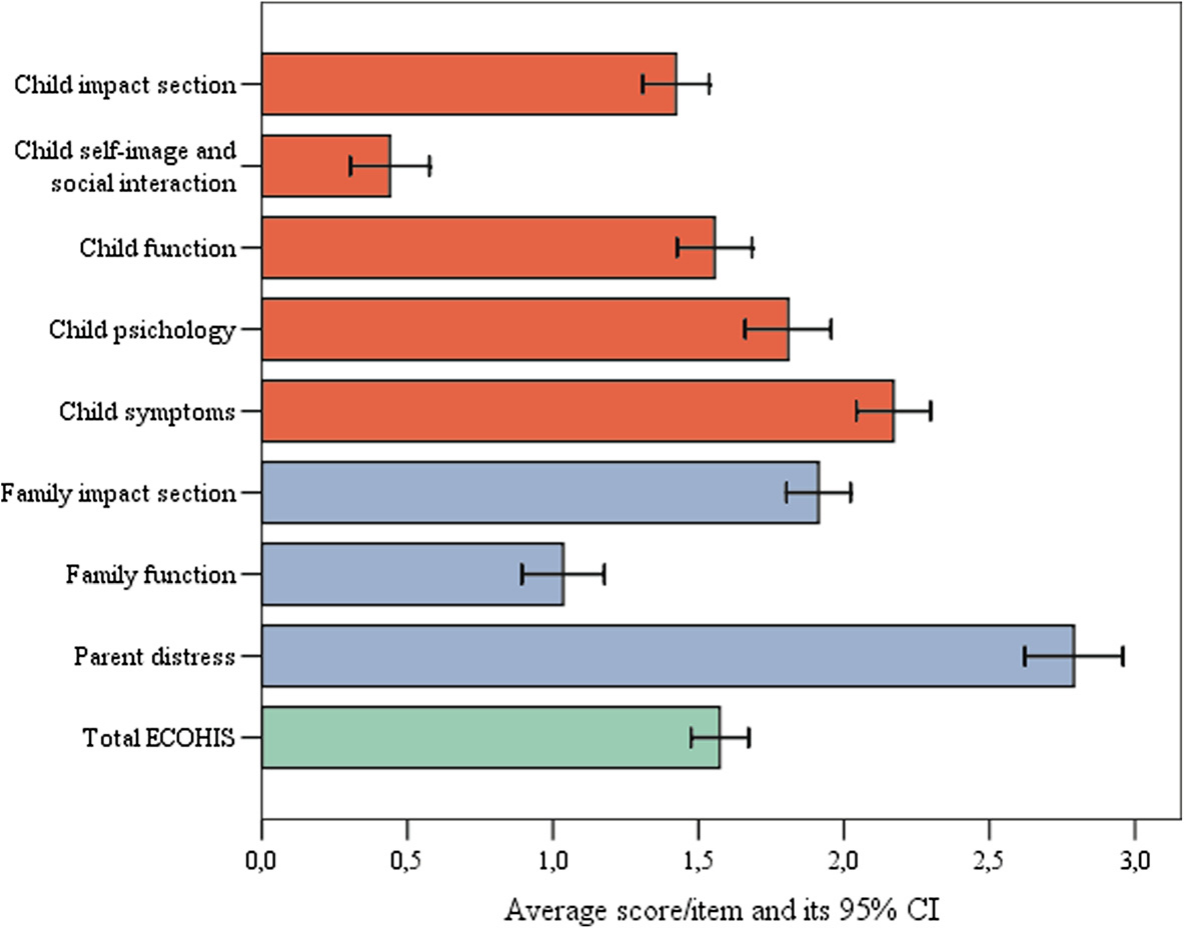
Mean overall and domain scores in the ECOHIS at baseline (N = 140).

Table 1 shows data on the cross-sectional construct validity. Parents who rated their child’s well-being as more severely affected reported higher child impacts. As for the family impact section, the mean score for those reporting “a lot” was slightly lower than those reporting “some”. Cronbach’s alphas for the total ECOHIS, as well as the child and family impact sections were 0.80, 0.80 and 0.46, respectively.Figure 1 illustrates the pre-treatment scores of total ECOHIS and its domains. The parents reported more family impacts than child impacts. The domain of parental distress had the highest score, whereas the score for the social wellbeing domain was the lowest of the ECOHIS domains.

**Table 1:** Baseline ECOHIS scores across the global rating categories

	**How much does the condition of your child’s teeth, lips, jaws or mouth affect his/her overall wellbeing?**				
	**Not at all**	**Some**	**A lot**	**Very much**	**P-value**
Number of patients (%)	3 (2.1)	22 (15.7)	65 (46.4)	50 (35.7)	-
Total ECOHIS	9.0 (2.7)	15.0 (4.4)	20.0 (7.0)	25.0 (8.3)	<0.001
Child impact section	3.0 (1.0)	7.4 (2.0)	12.8 (5.1)	15.8 (6.7)	<0.001
Child symptoms	0.7 (0.6)	0.9 (0.6)	2.4 (0.9)	2.3 (1.1)	<0.001
Child functions	2.0 (1.0)	4.3 (1.9)	5.9 (2.8)	7.7 (3.2)	<0.001
Child psychology	0.3 (0.6)	2.2 (1.6)	3.7 (1.6)	4.4 (1.5)	<0.001
Child self-image and social interaction	0.0 (0.0)	0.0 (0.0)	0.8 (1.4)	1.4 (2.1)	0.006
Family impact section	6.0 (2.0)	7.2 (1.7)	6.8 (2.5)	9.1 (2.6)	<0.001
Parent distress	4.3 (4.0)	5.5 (1.9)	5.1 (2.2)	6.3 (1.5)	0.007
Family function	1.7 (2.1)	1.7 (0.9)	1.7 (1.4)	2.7 (2.0)	0.004

Values are mean scale score (brackets contain standard deviation unless otherwise indicated).

The ECOHIS scores were associated with patient gender, age and parental education level (Table 2). The parents reported greater impacts on boys than on girls. Older children (4–6 years) experienced more pain than did younger ones, but the impact on the family was greater if the child was younger than four years. Children with highly educated parents had lower scores in the child impact section (3 of 4 domains).

**Table 2:** Pre-treatment ECOHIS scores by patients’ background

**Number of patients**	**Gender**		**Age (years)**		**Parental education**^**a**^	
	**Male**	**Female**	**<4**	**4-6**	**High**	**Other**
	**75**	**65**	**74**	**66**	**68**	**72**
Total ECOHIS score	21.7 (8.5)	18.9 (6.6)*	20.5 (9.2)	20.4 (5.8)	18.6 (6.2)	22.2 (8.6)*
Child impact section	13.7 (6.8)	11.8 (5.2)	12.4 (7.2)	13.2 (4.9)	10.8 (4.5)	14.7 (6.9)**
Child symptoms	2.1 (1.1)	2.1 (1.1)	1.9 (1.2)	2.3 (1.0)*	1.8 (1.0)	2.4 (1.1)*
Child functions	6.7 (3.3)	5.6 (2.7)*	6.0 (3.5)	6.5 (2.6)	5.6 (2.6)	6.8 (3.4)*
Child psychology	3.8 (1.9)	3.5 (1.5)	3.5 (1.8)	3.8 (1.7)	3.0 (1.5)	4.2 (1.8)**
Child self-image and social interaction	1.1 (1.8)	0.6 (1.4)	0.9 (1.8)	0.7 (1.4)	0.4 (0.7)	1.3 (2.1)*
Family impact section	8.0 (2.7)	7.2 (2.6)*	8.1 (2.8)	7.0 (2.3)*	7.8 (2.4)	7.5 (2.9)
Parent distress	5.7 (1.9)	5.4 (2.1)	5.8 (2.2)	5.3 (1.8)	5.8 (2.1)	5.4 (1.9)
Family function	2.3 (1.9)	1.7 (1.3)*	2.3 (1.9)	1.7 (1.1)*	2.0 (1.6)	2.1 (1.7)

Values are mean scale score (brackets contain standard deviation).

^a^High = University; Other = College or secondary.

*p < 0.05; Independent samples *t*-test.

**p < 0.001; Independent samples *t*-test.

Table 3 presents data on changes in ECOHIS scores from baseline to follow-up. The total ECOHIS and its subscale scores decreased significantly after the DGA treatment, demonstrating large effect sizes. Social wellbeing was the only domain, which demonstrated moderate effect size. The greatest decreases in scores were for the domains of child symptoms and child psychology in the child section and for the domain of parental distress in the family impact section.

**Table 3:** The mean ECOHIS domain scores at baseline and follow-up with effect sizes (N = 122)

**ECOHIS domain ** **(nr of items)**	**Baseline**		**Follow-up**		**Change in score (SD)**	**P-value**	**Effect size**
	**Mean (SD)**	**Range**	**Mean (SD)**	**Range**			
Total Ecohis score (13)	21.3 (6.9)	9-37	6.5 (4.8)	0-20	14.8 (7.9)	<0.001	2.1
Child impact section (9)	13.3 (5.6)	3-25	4.4 (3.7)	0-16	8.9 (6.2)	<0.001	1.6
Child symptoms (1)	2.1 (1.1)	0-4	0.4 (0.7)	0-2	1.8 (1.2)	<0.001	1.6
Child function (4)	6.5 (2.8)	0-13	3.1 (2.8)	0-11	3.5 (3.6)	<0.001	1.25
Child psychology (2)	3.7 (1.6)	1-6	0.8 (1.0)	0-3	2.9 (1.9)	<0.001	1.8
Child self-image and social interaction (2)	0.9 (1.6)	0-7	0.2 (0.6)	0-3	0.7 (1.7)	<0.001	0.4
Family impact section (4)	7.9 (2.4)	2-13	2.1 (1.9)	0-6	5.8 (2.7)	<0.001	2.4
Parent distress (2)	5.8 (1.8)	1-8	1.9 (1.9)	0-6	3.9 (2.2)	<0.001	2.2
Family function (2)	2.1 (1.7)	0-6	0.2 (0.6)	0-2	1.9 (2.6)	<0.001	1.1

Prevalence of the most frequently reported child and family impacts at baseline and follow-up are presented in Table 4. Pain, eating problems and feeling irritated were the most frequently reported impacts for children, whereas parents feeling upset and guilty were the most common impacts in the family section at baseline. Eating, pronunciation problems and parents feeling guilty were the most frequently reported impacts at follow-up. The biggest decrease in prevalence after the treatment was observed for the items of parents feeling upset and guilty.

**Table 4:** Prevalence of the most frequently reported impacts at baseline and follow-up (N = 122)

**Item**	Prevalence of impacts reported ‘often’ or ‘very often’*		
	**Baseline**	**Follow-up**	P-value**
Pain in the teeth mouth and jaws	44.3	0	<0.001
Difficulty drinking hot or cold beverages	33.6	3.3	<0.001
Difficulty eating some foods	59	26.2	<0.001
Difficulty pronouncing any words	16.4	18	>0.05
Missing preschool, daycare or school	8.2	0	<0.05
Trouble sleeping	23.8	0	<0.001
Being irritable or frustrated	38.5	0	<0.001
Avoided smiling or laughing	3.3	3.3	-
Avoided talking	0	0	-
Parents being upset	81.1	7.4	<0.001
Parents feeling guilty	73.8	12.3	<0.001
Parents taking time off from work	13.1	0	<0.001
Financial impact on the family	9	0	<0.001

*Values are percentage of parents, reporting the impact ‘Often’ or ‘Very often’.

**McNemar test.

More than half of the respondents (57%) rated their child’s oral health as good to excellent after DGA treatment, whereas 84% of them rated it as poor prior to the treatment. The majority of the parents (82%) reported that oral health status affected their child’s overall wellbeing considerably. More than half of them (54%) reported a substantial change in their child’s overall wellbeing after the treatment. All parents reported their satisfaction with the DGA treatment. Two-thirds of them (66.4%) felt they had received sufficient information prior to the treatment. More than half of the parents (64.8%) stated they would consider another DGA treatment, if needed.

## Discussion

This study presents new information about OHRQoL among young Lithuanian children prior to and after DGA treatment. Although young children and their families suffer greatly from the consequences of poor dental health, their OHRQoL improved significantly after DGA treatment.

The data were collected over the three-year period of this survey of young DGA patients treated in the LUHS Hospital, a tertiary-care treatment hospital in Kaunas, the second largest city in Lithuania. Although the results of this study do not represent the whole of Lithuania, our findings are likely to be close to those of the whole population, because the LUHS Hospital is the largest medical referral centre for all regions in the country, and any patient may choose this University Hospital when referred to DGA. The follow-up rate in our study was higher than in other similar studies [29]. The surprising main reason for dropping out of the study was the frequent emigration from Lithuania. The fact that the study sample at baseline included all child patients receiving DGA treatment in a three-year period and that only a few patients were lost in follow-up can be considered strength of the study. On the other hand, our study had no untreated control group which is usually required for such intervention studies, namely because dental treatment cannot be withheld from patients who need it. Findings on children’s OHRQoL reported by parents for very young children can be questionable, however this didn’t affect our results since just a minor fraction of the children (<10%) were younger than 3 years.

The magnitude of the impacts on OHRQoL in our study may partially stem from the method used: the ECOHIS was chosen as the one available for preschool-age children at the time of our study. Many of the other DGA studies used the Parental-Caregivers Perceptions Questionnaire and Family Impact Scale (P-CPQ & FIS), a much broader questionnaire (49 questions) designed for school-age children [16,18,19]. A shorter OHRQoL questionnaire (13 vs. 49 questions) in the ECOHIS seems to have advantages in evaluating young children’s quality of life, as found in the Dutch study, where both questionnaires were used [26]. The results of our and other DGA studies using ECOHIS revealed acceptable evaluative properties of the instrument [14,26]. Nevertheless, the ECOHIS has recently been found to have some limitations which could undermine its suitability for use with children affected by severe dental caries [30] when compared to the new short-form P-CPQ and FIS scales [7]. Our study showed low Cronbach alpha scores for the family section and for the item of financial problems as seen earlier [30]. Due to this and the different development process of the ECOHIS and the short-form P-CPQ and FIS scales [6,7], the ECOHIS might be better deployed in epidemiological surveys, and the short-form P-CPQ and FIS would be preferable for work in clinical samples with high disease levels, as suggested by Thomson et al. [30].

The observed impact of poor dental status on the OHRQoL of the Lithuanian child patients before the DGA treatment was greater than that in other follow-up studies [14,17,26] using the same ECOHIS instrument. A high prevalence and experience of dental caries among preschool-age children, together with shortcomings in the oral health services in Lithuania may explain the high pre-treatment scores in our study.

The most commonly reported impacts in our sample were similar to those of previous DGA studies [14,24]. A systematic review reported that social wellbeing was the least affected domain [29] as seen in our study too. A possible reason for this finding may be the widely discussed limits to parents’ knowledge about the social aspects of a child’s OHRQoL [31].

The parents reported greater impacts on boys than on girls, though we found no gender differences in dental health status regarding our patients [23] or among Lithuanian preschool aged children [20]. No gender differences in impacts were observed in another DGA study by Klaassen et al. [26]. Psychological factors may have played a role, but confirming and explaining this finding will require further research.

The educated parents reported fewer child impacts than did parents with a lower level of education. This is an interesting finding that raises questions about different health values among parents with regard to their education level. In general population, a higher level of parental education is associated with better OHRQoL in children [32], but it may be different among parents of children with high levels of dental disease.

The effect sizes observed in our study were significantly higher than those of other studies: the percentile reduction in total ECOHIS score after DGA treatment was 69.5% vs. 27.6% in a study by Lee et al. [14] and 42.7% in one by Klaassen et al. [17]. These findings can partly be attributed to different follow-up times, but the high prevalence of caries and poor dental health among young Lithuanian children certainly plays a role also: the more serious the problem, the more evident the result after solving it.

Despite the considerable improvement after DGA in most aspects of OHRQoL, some of the patients continued experiencing problems related to their dental disease and the treatment performed. Earlier DGA studies have also acknowledged, though not emphasised, this finding [29]. DGA, though effective, does not eliminate the impacts of ECC; a functional limitation for the child as well as parental distress may persist. Multiple caries treatments and extractions in most DGA patients [23] may explain these findings.

In the present study a majority of parents rated their child’s oral health at baseline as poor, a fact that reflects severe dental problems and poor quality of life, and leads parents to favour dental treatment under GA. The proportion is markedly higher than in the Netherlands, for example, where parental ratings were less serious-minded (only 7% of Dutch parents rated their children’s oral health as poor) [17]. Two-thirds of the parents favoured undergoing another DGA treatment for their child in future, a fact not accepted by the health care professionals. Repeated DGA treatments should, in fact, be avoided; rather, parents and their children should be guided towards preventive dental care. The study suggests that the protocol for providing parents with sufficient information prior to DGA treatment ought to be revised. Despite their high level of satisfaction with the treatment performed, one-third of parents felt that they did not receive all the necessary information prior to the treatment, another fact which further advocates change in DGA treatment services in the LUHS Hospital.

## Conclusions

The OHRQoL of young Lithuanian children in need of DGA treatment is seriously impaired. Dental general anaesthesia treatment is associated with significant improvements in the children’s OHRQoL. The children’s parents greatly appreciate this treatment modality, which has a positive impact on the family’s quality of life.

## Abbreviations

ECC: Early childhood caries; DGA: Dental general anaesthesia; OHRQoL: Oral health-related quality of life; ECOHIS: Early childhood oral health impact scale; GA: General anesthesia; LUHS: Lithuanian University of Health Sciences; dmft: Decayed missing, and filled primary teeth; P-CPQ: Parental-caregivers perceptions questionnaire; FIS: Family impact scale; SD: Standard deviation; CI: Confidence interval.

## Competing interests

The author(s) declare that they have no competing interests.

## Authors’ contributions

BJ designed and carried out the study, performed the statistical analyses and drafted the manuscript. JV made substantial contributions to analysis and interpretation of data and was involved in drafting the manuscript. RK participated in designing the study, acquisition of data and revising the manuscript critically. JN contributed to the design of the study and helped to draft the manuscript. All authors read and approved the final manuscript.

## Pre-publication history

The pre-publication history for this paper can be accessed here:

http://www.biomedcentral.com/1472-6831/14/81/prepub
